# Predictive Value of Inflammatory Biomarkers in Assessing Major Depression in Adults

**DOI:** 10.3390/biomedicines12112501

**Published:** 2024-10-31

**Authors:** Radu Gavril, Petru Romeo Dobrin, Alin Constantin Pînzariu, Mihaela Moscalu, Radu Gheorghe Grigore, Vlad Teodor Iacob, Andreea Cristina Bejenariu, Elena Rodica Popescu, Raluca Gavril, Bogdan Gireadă, Radu Petru Soroceanu, Ovidiu Gavrilovici, Cristinel Ștefănescu

**Affiliations:** 1 Department of Medicine III, Faculty of Medicine, “Grigore T. Popa” University of Medicine and Pharmacy of Iasi, 700115 Iasi, Romania; 2 “Socola” Institute of Psychiatry, 36 Bucium Street, 700282 Iasi, Romania; 3 Department of Morpho-Functional Sciences II, “Grigore T. Popa” University of Medicine and Pharmacy, 700115 Iasi, Romania; 4 Department of Preventive Medicine and Interdisciplinarity, “Grigore T. Popa” University of Medicine and Pharmacy, 700115 Iasi, Romania; 5 Faculty of Psychology and Education Sciences, “Alexandru Ioan Cuza” University, 700554 Iasi, Romania; ierei.zenovie10@yahoo.com (R.G.G.); gavrilov@uaic.ro (O.G.); 6 Department of Surgery I, “Grigore T. Popa” University of Medicine and Pharmacy, 700115 Iasi, Romania

**Keywords:** inflammation, depression, major depressive disorder, inflammatory markers

## Abstract

**Background:** There are studies that have investigated the association of pro-inflammatory cytokines with depressive disorders, but they often present certain limitations. In this study, two substantial groups of patients were analyzed: 92 patients with major depressive disorder and 76 without depressive disorders. The strict inclusion and exclusion criteria for the analyzed groups significantly increased the value of the obtained results. The research question of this study was whether levels of inflammation, measured by the inflammatory markers IL-6, IL-1α, and TNF-α, could predict the severity of depressive symptoms. This could provide additional evidence supporting the hypothesis that inflammation plays a notable role in the pathogenesis of depression. The data analysis supports the hypothesis that the biological mechanisms of inflammation contribute to the clinical manifestations of depression. Elevated levels of inflammatory markers, especially interleukins (IL-6, IL-1α) and tumor necrosis factor-alpha (TNF α), have been identified in patients with major depressive disorder compared to the findings in healthy controls. **Materials and Methods:** Inflammatory markers (IL-6, IL-1α, and TNF-α) were measured in a sample of 92 patients hospitalized at the Socola Institute of Psychiatry in Iasi, Romania, and compared to a control group with no depression or inflammatory conditions (n = 76). Severity of depressive symptoms was assessed using HAM-D scores. **Results:** The study results indicated that values of plasma inflammatory markers were significantly higher in patients with major depressive disorder (MDD) compared to the control group (IL-1α: 1.16 ± 0.44 pg/mL vs. 0.89 ± 0.25 pg/mL, *p* = 0.0004; IL-6: 9.21 ± 4.82 pg/mL vs. 7.16 ± 4.32 pg/mL, *p* = 0.0149; and TNF-α: 2.02 ± 0.96 pg/mL vs. 1.67 ± 0.8 pg/mL, *p* = 0.0286). The differences remained significant after applying logarithmic transformation, which was necessary to adjust for outlier values. An analysis of demographic characteristics showed that the frequency of women (67.4% vs. 36.84%, *p* < 0.001), cohabiting individuals (28.26% vs. 10.53%, *p* = 0.0001), and alcohol consumers (67.39% vs. 47.37%, *p* = 0.0087) was significantly higher in patients with MDD. The level of education was significantly lower in patients with MDD (median (IQR): 12 (2.5) years vs. 14 (8) years, *p* = 0.0016). The evaluation of confounding variables, including patients’ gender, marital status, education level, and alcohol consumption, was performed using multiple linear regression models. The results indicated that these demographic variables did not significantly influence the correlation between the HAM-D score and the values of IL-6, IL-1α, and TNF-α. A significant correlation between the HAM-D score and the logarithmic values of inflammatory markers was observed for log IL-1α in men (r = 0.355, *p* = 0.0014), log IL-6 in women (r = 0.0313, *p* = 0.0027), and log TNF-α in women (r = 0.3922, *p* = 0.0001). The results of the multiple linear regression and predictive analysis indicated that IL-1α (AUC = 0.677, *p* = 0.0004), IL-6 (AUC = 0.724, *p* < 0.001), and TNF-α (AUC = 0.861, *p* < 0.001) demonstrate high accuracy in discriminating patients with MDD. **Conclusions:** The results highlighted that IL-6 (AUC = 0.724; 95% CI: 0.648–0.801) and TNF-α (AUC = 0.861; 95% CI: 0.797–0.925) are significant predictors for major depressive disorder. The study highlights the potential of cytokines (IL-1α, IL-6 and TNF-α) as diagnostic markers. These findings support the hypothesis that inflammation may play an important role in the development or exacerbation of depressive symptoms.

## 1. Introduction

There is a multitude of factors that can determine a bidirectional relationship between depression and inflammation, with the underlying mechanisms not being fully understood [1]. Experimental studies have shown that stress is associated with elevated levels of CRP and TNF-α [2], that a stressful social family environment can directly stimulate the production of pro-inflammatory cytokines, and that changes in coagulation factors and genetic factors can increase the risk of depressive symptoms as well as cardiovascular diseases and glucose metabolism alterations, considering the theory that the inflammatory process is an etiological factor in depression associated with increased levels of inflammatory markers [3].

The inflammatory theory of depression is supported by recent research that indicates a high correlation between inflammatory processes and the emergence of major depressive disorder in adults [4]. Neurochemical imbalances and impaired synaptic plasticity, biological mechanisms linked to the pathophysiology of depression, may be exacerbated by impairments of glial cells, including astrocytes and changes in inflammatory markers, such as IL-1β, which induces mesencephalic progenitor cells in rats to transform into a dopaminergic phenotype, and IL-6, which decreases the survival of serotonergic neurons in the fetal brain [4,5,6].

Animal models have provided evidence supporting the inflammatory hypothesis of depression in recent research, highlighting a strong connection between inflammatory processes and various environmental and physical factors. These factors contribute to subtle differences in neuropsychiatric characteristics. Nonetheless, across these diverse depression models, it was identified that shared genetic alterations linked to increased neuroinflammation and synaptic dysfunction [7].

In this study, we aimed to more precisely assess the association between inflammation and depression by measuring inflammatory markers (IL-6, IL-1α, and TNF-α). A primary objective was to determine whether there is a significant correlation between inflammation levels, measured by inflammatory markers (IL-6, IL-1α, and TNF-α), and the severity of depressive symptoms, which were evaluated using HAM-D scores. This could provide evidence for the hypothesis that inflammation may play a role in depression. Additionally, the relationship between inflammation and depression was thoroughly evaluated.

This could provide insights into how social or clinico-demographics interact with inflammatory processes in depression.

The main question is whether plasma concentrations of pro-inflammatory cytokines (IL-6, IL-1α, and TNF-α) can serve as predictive biomarkers for depression severity and whether these concentrations are modulated by gender among patients diagnosed with major depressive disorder (MDD).

Therefore, this study aims to compare inflammatory markers across two distinct patient groups to assess the potential association between cytokine levels (IL-6, IL-1α, and TNF-α) and the severity of depressive symptoms. The overarching goal is to test the hypothesis that the intensity of depression can be predicted based on these specific inflammatory markers.

## 2. Materials and Methods

### 2.1. Subjects

A cross-sectional study was conducted, involving patients evaluated between June 2023 and September 2024 at the Socola Institute of Psychiatry. From the 769 patients consulted, 168 were included in the study and divided into two groups: the study group and the control group. All participants provided informed consent. This study was approved by the Ethics Committee of the Socola Institute of Psychiatry (No. 25882/20.08.2024).

**Figure 1:**
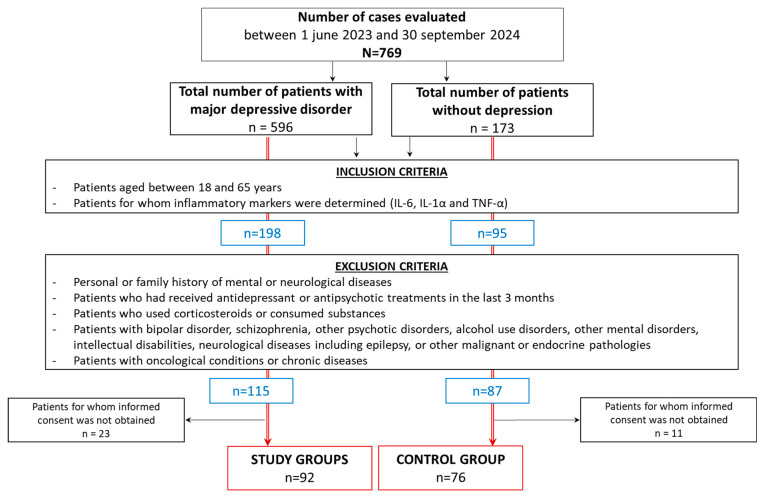
Flow chart of the selection of the study groups.

The general inclusion criterion was an age range of 18 to 65 years. A series of clinical and demographic characteristics, along with their associations with inflammatory markers (IL-6, IL-1α, and TNF-α), were analyzed for the subjects included in the study. The study group consisted of 92 patients with major depressive disorder (MDD), while the control group included 76 patients without depression and with no personal or family history of psychiatric or neurological diseases (Figure 1).

We used the Hamilton Depression Rating Scale (HAM-D) as the main psychometric tool to assess the severity of depression in patients. The Hamilton Depression Rating Scale is a reliable tool for assessing depression levels. This instrument was administered by clinically experienced psychiatrists who assessed participants’ psychiatric and medical history and confirmed their eligibility. The study group included adult patients with a HAM-D score of greater than 17. These patients were diagnosed with severe and very severe depression. The control group included patients with a HAM-D score of lower than 8 (without depression).

Patients who had received antidepressant or antipsychotic treatments in the previous 3 months were excluded from both groups. Additionally, patients using corticosteroids or other substances were not included in the study.

Patients with bipolar disorder, schizophrenia, or other psychotic disorders; alcohol use disorders; other mental disorders; intellectual disabilities; neurological diseases, including epilepsy; or other malignant or endocrine pathologies were also excluded.

The sample size was calculated based on the prevalence of depression. Globally, an estimated 5% of adults suffer from depression [8]. To calculate the minimum sample size that would ensure adequate representativeness of the patient category, we used the formula $n\geq {\left(Z_{\left(1-\frac{\alpha }{2}\right)}\right)}^{2}\times \frac{p\left(1-p\right)}{d^{2}}$, with Z = 1.96 for a 95% confidence interval and a “d” value corresponding to an estimation error of 5%. For a maximum assumed error of 5%, the minimum sample size should be 73 cases. For both patient groups included in the study, this condition was met.

### 2.2. Demographic and Clinical Data

The demographic data analyzed included patient age, gender, marital status, education (years of study), body mass index (BMI in kg/m^2^), smoking status, alcohol consumption, and living environment. The clinical parameters evaluated included the HAM-D (Hamilton Depression Rating Scale) to assess depression levels. The inflammatory biomarkers analyzed in the study were the concentrations of cytokines IL-6, IL-1α, and TNF-α. These were measured in serum samples using the ELISA method. Blood samples were taken between 8:00 and 9:00 in the morning. After 30 min of centrifugation at 8000 RPM, the plasma was separated, and the supernatant was kept for up to six months at −80 °C. As directed by the manufacturer, commercially available enzyme-linked immunosorbent assay (ELISA) kits (DuoSet, R&D Systems, Minneapolis, MN, USA) were used for the measurements of IL-6, IL-1α, and TNF-alpha. Every sample was tested twice, and the value of the declared probe was equal to the value of the duplicate probe. The assays’ detection criteria were 0.4 pg/mL for IL-6 and IL-1α and 25 pg/mL for TNF-alpha. IL-1α, IL-6, and TNF-alpha levels are expressed in pg/mL.

Patients with comorbid psychiatric disorders, such as bipolar disorder, schizophrenia, or schizoaffective disorder, were excluded to isolate the effects of MDD alone. Individuals with active substance use disorders were also excluded, as substance abuse can significantly impact the course of depression and treatment outcomes. Patients who had undergone electroconvulsive therapy (ECT) within the previous six months or who were currently receiving ECT were excluded to control for the effects of this potent intervention on depression severity. Additionally, those with significant medical conditions (e.g., neurological disorders, uncontrolled diabetes, or heart disease) that might interfere with psychiatric assessment or treatment adherence were also excluded to ensure that the results would specifically pertain to MDD without major medical complications (Figure 1).

### 2.3. Statistical Analysis

The variables of interest that were included in the study were processed statistically using SPSS v.29 (IBM Ireland Product Distribution Limited, IBM House, Shelbourne Road, Ballsbridge, Dublin, Ireland) and the STATA 16 software (StataCorp LLC, 4905 Lakeway Drive, College Station, TX, USA). Specific statistical indicators were used to describe the analyzed parameters, depending on the characteristics of the variable (type of variable, distribution of the series of values, homogeneity, etc.). The ANOVA test (one-way analysis of variance) and the Student *t* test was used to compare continuous variables with normal distribution, and the Mann–Whitney U test was used for data that did not meet the condition of normal distribution.

Qualitative variables were presented as absolute (n) and relative (%) frequencies, and comparison between groups was based on Chi-square test results. The Fisher’s exact test (superscript F) was used only if the Chi-square consistency was not met (small number of cases) (Fisher’s exact test is used as a replacement for the Chi-square test when the expected frequency of one or more cells is less than 5). Considering the fact that the distribution of inflammatory marker values was not normal and outlier values were identified, the logarithmic transformation of the series of values was imposed. Thus, outlier values were excluded. The comparison of the levels of biomarkers based on the ANOVA test included the assessment of the partial effect size (the partial eta squared (η^2^) effect sizes for one-way ANOVA). The partial eta squared effect sizes for one-way ANOVA were reported and classified as small (η^2^ = 0.01), medium (η^2^ = 0.06), or large (η^2^ =0.14).

The significance level calculated in the utilized tests (*p*-value), *p* < 0.05 was considered statistically significant.

## 3. Results

In the study group, which included patients with MDD, the mean age was 37.86 years (SD = 10.85). The average age of the study group did not differ significantly (*p* = 0.4286) compared to the control group (Table 1). The proportion of female patients in the study group (67.4%) was significantly higher (*p* < 0.001) than that in the control group (63.16%). The characteristics of the study participants are presented in Table 1. Significant differences were found between the groups in terms of marital status (*p* = 0.0001), education level (*p* = 0.0016), and alcohol consumption (*p* = 0.0087). There were no significant differences between the two groups regarding BMI (*p* = 0.1917), smoking status (*p* = 0.0650), or living environment (*p* = 0.9381).

The analysis of plasma inflammatory markers revealed significant differences for IL-1α (*p* = 0.0004), IL-6 (*p* = 0.0149), and TNF-α (*p* = 0.0286). Higher values of IL-1α, IL-6, and TNF-α were observed in the study group.

The concentrations of cytokines IL-6, IL-1α, and TNF-α did not exhibit a normal distribution, with extreme values being observed. Therefore, a logarithmic transformation was applied to all these series of values to reduce the skewness of the distributions.

The study analyzed whether there were significant differences between men and women regarding the relationship between inflammation and depression. This could contribute to understanding how a patient’s gender may influence this relationship. The results of the comparative analysis are presented in Table 2.

The analysis of demographic characteristics, clinical characteristics, and inflammatory markers according to the study group (the presence of HAM-D) did not reveal significant differences according to the gender of the patients. This aspect can be explained by the homogeneity of the cases included in the study groups. The study aimed to eliminate possible confounding variables, strictly respecting the inclusion and exclusion criteria in the batch.

However, the analysis noted in the study group the presence of statistically significant differences between women and men for smoking (*p* = 0.0345) and alcohol consumption (*p* = 0.0379) (Table 2).

The high values of the effect size (η^2^) indicate a strong relationship between HAM-D scores and the inflammatory biomarkers IL-1α, IL-6, and TNF-α (Table 3).

To evaluate whether there were variables that may influence the correlations between HAM-D scores and inflammatory biomarkers (IL-1α, IL-6, TNF-α), a series of multiple linear regression models were created to adjust for confounding factors. The standardized coefficients (β) and calculated significance levels (*p*-value) were interpreted. In the adjusted models, it was observed that the standardized coefficients calculated for the correlation between HAM-D and IL-1α, IL-6, and TNF-α did not change significantly. These results demonstrate that the confounding variables did not influence the correlation between HAM-D and these inflammatory biomarkers (Table 4).

**Figure 2:**
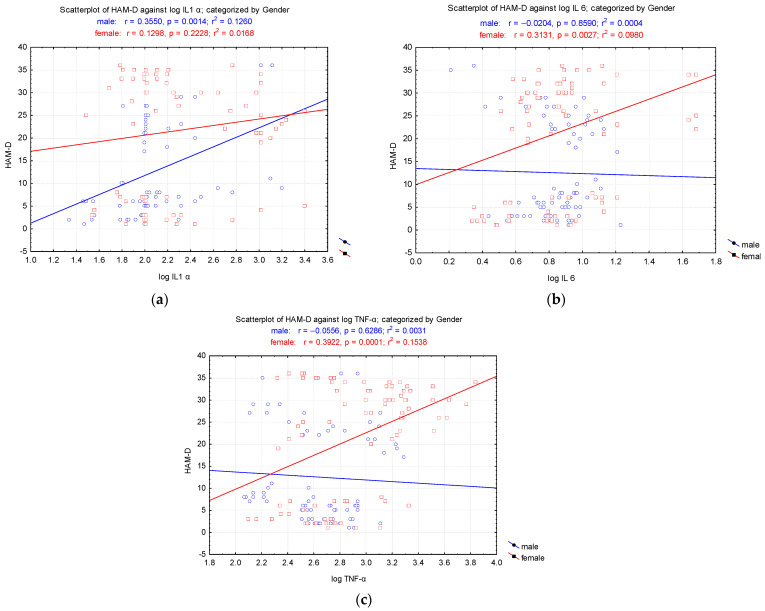
Pearson correlation of the pro-inflammatory cytokines interleukin 1 alpha (IL-1α), interleukin 6 (IL-6), and tumor necrosis factor alpha (TNF-α) vs. HAM-D by gender. (**a**) The correlation between IL-1α and HAM-D was significant for males but non-significant for females. (**b**) The correlation between IL-6 and HAM-D was significant for women but non-significant for men. (**c**) The correlation between TNF-α and HAM-D was significant for women but non-significant for men.

The results were analyzed for Pearson’s correlations of pro-inflammatory cytokines (IL-1α, IL-6, TNF-α) with psychometric measures per cohort, group (HAM-D and control), and sex (females and males) (Figure 2). The correlation analysis conducted separately for each group based on gender indicated a significant correlation between the HAM-D score and log IL-1α for male patients (r = 0.3550, *p* = 0.0014) and with IL-6 for female patients (r = 0.3131, *p* = 0.0027). TNF-α values showed a significant correlation with HAM-D only in female patients (r = 0.3922, *p* = 0.0001) (Figure 2).

The multivariate linear regression analysis (Table 5), with HAM-D scores as the dependent variable and the concentrations of cytokines IL-6, IL-1α, and TNF-α as independent variables, indicated that all analyzed pro-inflammatory markers were significantly associated with the HAM-D score.

**Figure 3:**
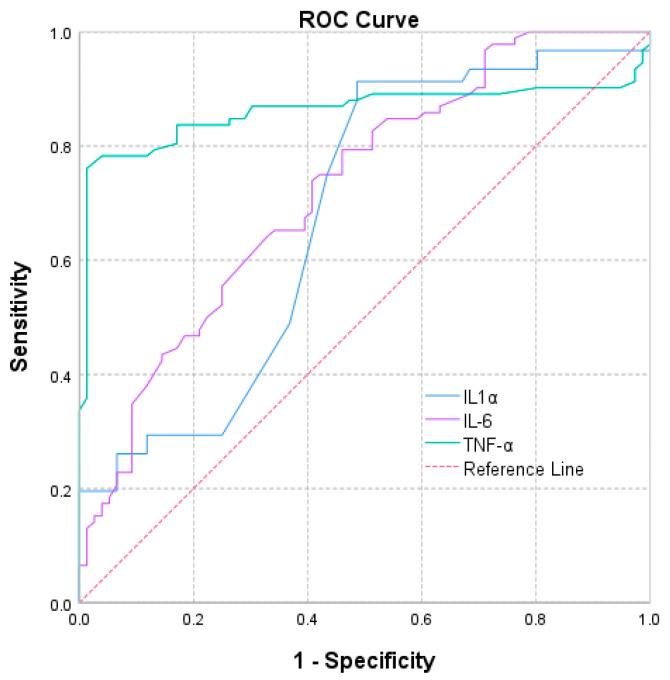
ROC curves of IL-1α, IL-6, and TNF-α.

The predictive value of the cytokines IL-1α, IL-6, and TNF-α in the discrimination of patients with severe depression was assessed based on the AUC values (Table 6), which is also presented graphically by the ROC curve (Figure 3).

As shown in Figure 3, ROC curve analysis demonstrated that IL-1α, IL-6, and TNF-α showed high accuracy in discriminating patients with severe depression.

The study highlights a significant correlation between the levels of inflammation and the severity of depressive symptoms, supporting the hypothesis that inflammation plays a crucial role in the development or exacerbation of depression. This correlation opens new avenues for anti-inflammatory therapies as potential treatments for depression patients resistant to traditional treatments.

## 4. Discussion

The relationship between inflammation and depression is also affected by risk and protective factors, which require further research. Lifestyle factors, such as diet and physical activity level, can influence inflammation levels and, consequently, the risk of developing or exacerbating depression [9].

As we better understand the connection between inflammation and depression, there is an opportunity to develop personalized therapies. A treatment that works effectively for one patient may not have the same effect for another, depending on their inflammation levels. Identifying these individual differences can lead to more precise and effective approaches to the treatment of depression [10].

Physicians and therapists ought to consider evaluating inflammation levels and integrating this information into the development of personalized treatment plans for patients with depression [11]. This could offer a more efficient and precise approach to managing and treating depression. In our study, the analysis of plasma inflammatory markers revealed significant differences for IL-1α (*p* = 0.0004), IL-6 (*p* = 0.0149), and TNF-α (*p* = 0.0286), with higher levels in the study group, and a logarithmic transformation was applied to the concentrations of IL-6, IL-1α, and TNF-α due to their non-normal distribution and extreme values.

In light of these complex aspects and the uncertainties surrounding the relationship between inflammation and depression, it is evident that this is a crucial area of research in psychiatry [12]. With a deeper understanding of these biological processes, we can develop more effective interventions for treating and managing depression, thus offering hope and help to those struggling with this debilitating mental disorder.

Some of these studies have shown that patients with depression may have elevated levels of these inflammatory markers compared to healthy individuals [13]. Moreover, positive correlations have been identified between the severity of depressive symptoms and high levels of these markers, suggesting that inflammation may contribute to the severity of depression [14]. Our study demonstrated that the correlation analysis conducted separately for each gender group showed a significant association between the HAM-D score and log IL-1α in male patients (r = 0.3550, *p* = 0.0014) and with IL-6 in female patients (r = 0.3131, *p* = 0.0027). Additionally, TNF-α values were significantly correlated with HAM-D scores only among female patients (r = 0.3922, *p* = 0.0001).

Challenges for future studies include balancing potential side effects and considering somatic comorbidities, especially cardiovascular diseases, and treatment risk [15]. Caution was highlighted in a recent study on the potential use of NSAIDs (non-steroidal anti-inflammatory drugs) in depression, emphasizing that NSAID effects are inconsistent due to methodological heterogeneity and highlighting the need for methodological improvements in future studies [16]. Additionally, other pathways for personalized treatment schemes, such as nitrosative and oxidative stress pathways and increasing glutathione levels, are important to consider [17]. Clearly, the widespread use of anti-inflammatory agents against depression is not indicated. Rather, the challenge lies in identifying patients who might effectively respond to anti-inflammatory intervention [18]. Regarding the clinical applicability of these results, they could suggest that measuring inflammation levels might be useful in evaluating and treating depression, especially in patients who do not respond well to traditional therapies. Anti-inflammatory therapies or interventions to reduce inflammation could be considered in the treatment of depression, particularly in cases where inflammation seems to play a significant role [18].

Studies have addressed the fundamental pathways through which cytokines can contribute to depression [19]. In general, it has been shown that cytokines access the brain and interact with almost every pathophysiological domain relevant to depression, including neurotransmitter metabolism, neuroendocrine function, and neuronal plasticity. However, it remains unclear whether the activation of inflammatory pathways in the central nervous system during depression originates mainly peripherally and/or whether stress or other processes not yet identified induce inflammatory responses directly in the brain [20].

Such unresolved issues will have a major impact on the fact that relevant therapeutic targeting will require activity at the CNS level to be effective [21]. The source of inflammation is clear when depression occurs in the context of medical conditions with an infectious, autoimmune, or inflammatory component or when there are injuries and/or tissue destruction, all of which are associated with the activation of the peripheral system and, in some cases, central inflammatory responses. In the case of depressive individuals with no apparent medical condition, the source of inflammation is less apparent, although developing inflammatory processes may be present [21].

Relevant data for the potential clinical applications of the association between inflammation and depression indicate that inflammatory biomarkers can identify depressive patients who are less likely to respond to conventional antidepressant treatment and can provide an indicator of treatment response. For example, patients with evidence of increased inflammatory activity before treatment have been reported to be less sensitive to antidepressants, lithium, or sleep deprivation (a strong short-term mood stimulant). Furthermore, it has been found that patients with a history of non-response to antidepressants demonstrate elevated plasma concentrations of IL-6 and acute-phase reactants compared to patients who respond to treatment [22,23]. Additionally, emerging literature suggests that functional allelic variants of genes for IL-1β and TNF-α, as well as genes critical for T-cell function, may increase the risk of depression and be associated with a reduced response to antidepressant therapy. Notably, antidepressant treatment has been associated with a reduction in inflammatory markers in 11 out of 20 studies that examined immune responses as a function of antidepressive therapy [24,25].

Depression, a severe psychiatric condition, represents a significant global challenge for public health [15]. Although the specialized literature extensively addresses the risk factors in the development of depression and identifies a series of inflammatory factors significantly associated with major depressive disorder (MDD), comprehensive studies are still needed to complete the profile of MDD patients. Additionally, studies are required to evaluate the response to conventional antidepressant treatment and to explore alternative supportive therapies. Inflammatory biomarkers are an important element in assessing the severity of MDD, but they can also evaluate the treatment response. A future perspective of this study is to present the results regarding the predictive value of inflammatory biomarkers in the response to MDD treatment. The combination of medication, psychotherapy, and somatic therapies remains an effective way to manage severe forms of depression. Furthermore, complex research is ongoing to personalize MDD therapies [18]. An increasing number of studies suggest that involvement in religious practices can provide individuals with a sense of belonging, shared beliefs, and social identity, which may be linked to improved mental well-being [24]. Religion also offers a framework for understanding one’s existence and for addressing existential questions. While examining a spiritually integrated program for those dealing with depression, a reduction in depressive symptoms from the beginning of the program to discharge was observed. It was found that religious comfort was negatively correlated with depression levels [25]. Gaining such insights into Christian views on mental health could highlight the role of social influence on help-seeking behaviors and the emphasis on spiritual solutions for this condition.

Another important aspect of this equation is the gender difference. Studies have suggested that the relationship between inflammation and depression may vary by sex, with potential implications for the clinical approach to depression in men and women [26,27]. For example, some research has indicated that women may exhibit a stronger correlation between inflammation and depression than men, suggesting that anti-inflammatory treatments might have a more beneficial effect among women with depression [27,28]. However, the relationship between inflammation and depression is far from fully understood. Ultimately, understanding the underlying mechanisms of the interaction between genetic, neurochemical, and inflammatory factors in the context of depression is essential for developing more effective and personalized therapeutic strategies for patients with depressive disorders, including those at increased risk of suicide. This knowledge can contribute to improving the quality of life and prognosis for these patients and can open new directions for research in psychiatry [29].

Many questions remain unanswered, including the exact mechanisms by which inflammation can influence brain function and, consequently, mental well-being. Additionally, questions arise about the causal direction of this relationship: Does inflammation cause depression, or vice versa, or is there a vicious cycle where these two processes influence each other?

Thus, the relationship between inflammation and depression is a fascinating and complex research area that has shed light on new directions in understanding and clinically approaching depression. Although there are many unanswered questions and much future research needed, this connection offers hopes for developing new and personalized therapies for patients with depression, thereby opening doors to improving the mental well-being of millions of people.

The results of this study provide a compelling argument for the significant correlation between inflammation levels, specifically the inflammatory markers IL-1α and IL-6, and the severity of depressive symptoms as assessed by HAM-D scores. These findings support the hypothesis that inflammation may play a crucial role in the development or exacerbation of depressive symptoms. This opens new avenues for research into anti-inflammatory therapies as potential treatments for patients with depression that is resistant to traditional treatment methods [30].

A key aspect highlighted by this study is the gender-based differences in the correlation between inflammation and depression. Women exhibited stronger correlations between inflammatory markers and depressive symptoms compared to men. This suggests that the underlying mechanisms of depression may differ between sexes, necessitating further research to fully understand these differences and develop personalized treatment approaches tailored to each gender [31].

Some studies support that divorced patients showed a significant positive correlation between IL-6 levels and depressive symptoms, whereas married patients did not exhibit the same significant correlation. This may indicate that stress factors associated with divorce could intensify the link between inflammation and depression [32].

Based on our results, a more comprehensive approach could involve evaluating the genetic component that may play a role in an individual’s susceptibility to this complex interaction [33]. Proximal factors with an underlying immune component can induce a sustained immune response, which modulates various downstream effectors, including monoamine metabolism, tryptophan metabolism, and activation of the hypothalamic–pituitary axis [34].

Overall, the findings suggest a complex interplay between neurochemical and inflammatory factors in the development and manifestation of depressive disorders. The correlation between increased activity of pro-inflammatory cytokines and the onset of depressive symptoms supports the inflammation–depression hypothesis. This indicates that treatments targeting inflammation reduction may be beneficial for patients with depression, especially those who do not respond to conventional antidepressant therapy [35].

## 5. Conclusions

The study highlights a significant correlation between inflammation levels, particularly the inflammatory markers IL-1α, IL-6, and TNF-α, and the severity of depressive symptoms. These findings support the hypothesis that inflammation may play an important role in the development or exacerbation of depressive symptoms. This correlation opens new avenues for anti-inflammatory therapies as potential treatments for patients with depression resistant to traditional treatments.

The results highlighted that IL-6 (AUC = 0.724; 95% CI: 0.648–0.801) and TNF-α (AUC = 0.861; 95% CI: 0.797–0.925) are significant predictors for major depressive disorder. The study shows the potential of cytokines (IL-1α, IL-6, and TNF-α) as diagnostic markers, highlighting the need for further research to develop personalized treatment approaches and identify biomarkers for estimating the response to anti-inflammatory therapies.

## Figures and Tables

**Table 1:** Demographic and clinical characteristics.

Characteristics	Study Group n = 92	Control Group n = 76	*p*-Value
Demographic data			
Age (years), mean (SD)	37.86 (10.85)	39.16 (10.2)	0.4286 ^†^
Gender, female/male, n (%)	62/30 (67.4/32.6)	28/48 (36.84/63.16)	<0.001 ^†^
BMI (kg/m^2^), mean (SD)	25.88 (4.42)	24.98 (4.43)	0.1917 ^†^
Marital status, n (%) Married Divorced Cohabitation	57 (61.96) 9 (9.78) 26 (28.26)	68 (89.47) 0 (0) 8 (10.53)	0.0001 ^‡^
Education (years), median (IQR)	12 (10–12.5)	14 (10–18)	0.0016 ^‡^
Smoking status (Yes/No), n (%)	68/24 (73.91/26.09)	65/11 (85.53/14.47)	0.0650 ^‡^
Alcohol consumption (Yes/No), n (%)	62/30 (67.39/32.61)	36/40 (47.37/52.63)	0.0087 ^‡^
Environment (rural/urban), n (%)	60/32 65.22/34.78)	50/26 (65.79/34.21)	0.9381 ^‡^
Clinical data			
HAM-D, median (IQR)	30 (28–34)	4.5 (2–6.5)	<0.001 ^§^
Plasma inflammatory markers			
IL-1α (pg/mL), mean (SD)	1.16 (0.44)	0.89 (0.25)	0.0004 ^†^
Log IL-1α (pg/mL), mean (SD)	2.31 (0.43)	2.05 (0.38)	0.0253 ^†^
IL-6 (pg/mL), mean (SD)	9.21 (4.82)	7.16 (4.32)	0.0149 ^†^
Log IL-6 (pg/mL), mean (SD)	0.92 (0.28)	0.81 (0.20)	0.00005 ^†^
TNF-α (pg/mL), mean (SD)	2.02 (0.96)	1.67 (0.81)	0.0286 ^†^
Log TNF-α (pg/mL), mean (SD)	2.93 (0.48)	2.63 (0.28)	0.0425 ^†^

(^§^) Mann–Whitney U test; (^†^) ANOVA test; (^‡^) Pearson Chi-square test or F—Fisher’s exact test.

**Table 2:** Comparison of plasma inflammatory markers and demographic characteristics between groups according to gender (n = 168).

Characteristics	Study Group (n = 92)		*p*-Value	Control Group (n = 76)		*p*-Value
	Female n = 62	Male n = 30		Female n = 28	Male n = 48	
Demographic data						
Age (years), mean (SD)	37.21 (10.38)	39.20 (11.84)	0.3985 ^†^	40.71 (12.15)	38.25 (8.89)	0.3284 ^†^
BMI (kg/m^2^)	25.57 (4.43)	26.51 (4.38)	0.3372 ^†^	24.52 (4.31)	25.24 (4.50)	0.4900 ^†^
Marital status, n (%) Married/divorced/cohabitation	39/3/20 (62.9/4.84/32.26)	18/6/6 (60/20/20)	0.0535 ^‡^	24/0/4 (85.71/0/14.29)	44/0/4 (91.67/0/8.33)	0.4147 ^‡^
Education (years), median (IQR)	11.89 (2.67)	12.34 (2.96)	0.5662 ^‡^	14.57 (4.38)	13.87 (4.09)	0.4027 ^‡^
Smoking status (Yes/No)	12/50 (19.35/80.65)	12/18 (40/60)	0.0345 ^‡^	4/24 (14.29/85.71)	7/41 (14.58/85.42)	0.9716 ^‡^
Alcohol consumption (Yes/No)	16/46 (25.81/74.19)	14/16 (46.67/53.33)	0.0379 ^‡^	17/11 (60.71/39.99)	23/25 (47.92/52.8)	0.2797 ^‡^
Environment (rural/urban)	20/42 (32.26/67.74)	12/18 (40/60)	0.4648 ^‡^	11/17 (39.29/60.71)	15/33 (31.25/68.75)	0.4762 ^‡^
Clinical data						
HAM-D, median (IQR)	30.91 (2.87)	30.50 (3.53)	0.4717 ^§^	3.28 (2.16)	3.34 (1.67)	0.9390 ^§^
Plasma inflammatory markers						
IL-1α (pg/mL), mean (SD)	1.21 (0.52)	1.08 (0.53)	0.1353 ^§^	0.93 (0.52)	0.87 (0.41)	0.4862 ^§^
Log IL-1α (pg/mL), mean (SD)	2.38 (0.46)	2.15 (0.29)	0.0896 ^§^	2.11 (0.39)	2.02 (0.37)	0.8229 ^§^
IL-6 (pg/mL), mean (SD)	9.47 (3.32)	8.67 (3.23)	0.4724 ^§^	7.83 (3.27)	6.78 (3.21)	0.0825 ^§^
Log IL-6 (pg/mL), mean (SD)	0.93 (0.31)	0.86 (0.24)	0.1820 ^§^	0.76 (0.27)	0.83 (0.22)	0.2238 ^§^
TNF-α (pg/mL), mean (SD)	2.03 (0.32)	1.99 (0.26)	0.3895 ^§^	1.67 (0.17)	1.65 (0.17)	0.6841 ^§^
Log TNF-α (pg/mL), mean (SD)	3.04 (0.37)	2.69 (0.39)	0.2519 ^§^	2.68 (0.31)	2.60 (0.26)	0.3552 ^§^

(^§^) Mann–Whitney U test; (^†^) Student’s *t*-test; (^‡^) Pearson Chi-square test or F—Fisher’s exact test.

**Table 3:** Determination of effect size. The value of eta squared (η^2^)—measure of the proportion of variation in the analyzed variables.

Study Group vs. Control Group	F	*p*-Value	Partial Eta-Squared (η^2^)
IL-1α	3.13078	0.000387	0.074130
IL-6	5.11975	0.014967	0.030273
TNF-α	1.96170	0.028671	0.021674
Log IL-1α	5.09409	0.025328	0.092141
Log IL-6	7.15186	0.000055	0.094682
Log TNF-α	1.64599	0.042520	0.073221

ANOVA test.

**Table 4:** Multiple linear regression coefficients for the models used to evaluate confounding variables.

Multiple Linear Regression Dependent Variable: HAM-D	Unstandardized Coefficients		Standardized Coefficients	*t*	*p*-Value
	B	Std. Error	β		
Model 1	Model summary: R = 0.324, R^2^ = 0.1049; ANOVA: F = 8.965, *p* = 0.0284				
IL-1α	5.024	0.564	0.211	3.241	0.019
Model 1a					
IL-1α	5.008	0.413	0.204	4.528	0.013
Gender, female	1.006	0.356	0.095	1.543	0.069
Marital status, Cohabitation	1.164	0.234	0.102	1.231	0.094
Education (years)	0.841	0.789	0.097	0.947	0.078
Alcohol consumption	0.754	0.567	0.089	0.856	0.085
Model 2	Model summary: R = 0.438, R^2^ = 0.191; ANOVA: F = 5.871, *p* = 0.0296				
IL-6	2.318	0.301	0.464	5.997	<0.001
Model 2a					
IL-6	2.259	0.384	0.486	5.786	<0.001
Gender, female	0.674	0.471	0.037		0.063
Marital status, Cohabitation	0.986	0.699	0.051		0.086
Education (years)	0.418	0.321	0.094		0.096
Alcohol consumption	0.321	0.148	0.084		0.124
Model 3	Model summary: R = 0.399, R^2^ = 0.159; ANOVA: F = 6.871, *p* = 0.377				
TNF-α	8.874	1.002	0.672	9.391	<0.001
Model 3a					
TNF-α	8.541	1.224	0.615	0.974	0.087
Gender, female	1.541	0.984	0.056	0.657	0.124
Marital status, Cohabitation	1.231	0.897	0.079	0.841	0.096
Education (years)	0.564	0.514	0.047	1.064	0.071
Alcohol consumption	0.754	0.754	0.048	1.134	0.098

**Table 5:** Multiple linear regression coefficients regarding changes in inflammatory markers in relation to the HAM-D depression score.

Multiple Linear Regression Dependent Variable: HAM-D	Unstandardized Coefficients		Standardized Coefficients	*t*	*p*-Value
	B	Std. Error	β		
(Constant)	−36.259	4.751		−7.632	<0.001
IL-1α	4.326	1.917	0.135	2.257	0.025
IL-6	1.122	0.271	0.245	4.141	<0.001
TNF-α	21.459	2.435	0.532	8.812	<0.001

Model summary: R = 0.667, R^2^ = 0.445; ANOVA: F = 43.837, *p* < 0.001.

**Table 6:** Predictive value (accuracy in prediction) a of inflammatory markers for severe depression.

Plasma Inflammatory Markers	Area Under the Curve (AUC)	Std. Error	*p*-Value	95%Confidence Interval for AUC	
				Lower Bound	Upper Bound
IL-1α	0.677	0.043	0.0004	0.594	0.761
IL-6	0.724	0.039	<0.001	0.648	0.801
TNF-α	0.861	0.033	<0.001	0.797	0.925

## Data Availability

The data presented in this study are available on request from the corresponding authors.
